# Comparison of Temperament and Character in Major Depressive Disorder Versus Bipolar II Disorder

**Published:** 2014

**Authors:** Sara Bensaeed, Atefeh Ghanbari Jolfaei, Farhad Jomehri, Alireza Moradi

**Affiliations:** 1 Department of Clinical Psychology, School of Psychology, Science & Research Branch, Islamic Azad University, Tehran, Iran.; 2 Minimally Invasive Surgery Research Center AND Department of Psychiatry, School of Behavioral Sciences and Mental Health, Iran University of Medical Sciences, Tehran, Iran.; 3 Department of Psychology, School of Psychology, Allameh Tabatabii University, Tehran, Iran.; 4 Department of Psychology, School of Psychology, Tarbiyat Moalem University, Tehran, Iran.

**Keywords:** Bipolar II Disorder, Major Depressive Disorder, Temperament and Character

## Abstract

**Objective:** The aim was to determine how personality of major depressive disorder (MDD) patients is different from that of bipolar II disorder (BIID) patients.

**Methods:** In this cross-sectional study, two groups of patients with MDD (47 patients) and BIID (45 patients) between 18 and 55 years old were included and compared. The research instrument that subjects answered to was Temperament and Characteristic Inventory-125-R.

**Results: **Among temperament dimensions, novelty seeking, and reward-dependently in contrast with other traits such as harm avoidance and persistence showed a significant difference between the two studied groups. Among characteristic dimensions, self-direction and self-transcendence demonstrated a significant difference between the two groups (p < 0.005).

**Conclusion:** Patients suffering from BIID are sensation seeker and are motivated by stimulates more often than MDD patients are. They feel euphoria more and, find the world more stimulating.

## Introduction

It is found in many studies that some of the personality traits are considered more important in mood disorders occurrence than others. Accordingly, it seems that people with some specific individual personality are more vulnerable to a specific type of mood disorders (1).

About 31 million US$/year is the expense imposed on US healthcare system in terms of industrial power related to major depressive disorder (MDD) (2). On the other hand, bipolar disorder is one of the prevalent, severe, and relapsing psychiatric disorders considered to be one of the major general health problems. There is potential relapse for over 90% of patients, and so it imposes high economic cost on families and societies (3).

Majority of bipolar II disorder (BIID) patients referred to psychiatric clinics are in depression phase; they do not show symptoms of hypomania, and therefore they might be diagnosed as MDD by psychiatrics (4). Hence, better identification of symptoms and differentiation between these two types of disorders is important in choosing a suitable treatment and preventive measures. Therefore, this research tried to introduce some new personality aspects of the above-mentioned disorders. This claim was followed by using Temperament and Characteristic Inventory (TCI).

The TCI is based on Cloninger’s psychobiological model of personality, which assumes that personality derives from the dynamic interaction among four temperament and three character traits (5). Temperament traits (novelty seeking [NS], harm avoidance [HA], reward dependence [RD], and persistence [P]) represent basic emotional responses, which are manifested early in life, are stable throughout life and are moderately heritable. Character traits (self-directedness [SD], cooperativeness [CO], and self-transcendence [ST]) represent concepts about self and personal relations, which are regulated by supervisory cognitive processes that develop throughout life (6).

Temperament involves a relatively small set of emotions associated with one’s basic need, for example, safety (so-called primary motives). Character refers to mind that is the conceptual core of personality and involves individual difference in self-concepts and object-relations that reflect personal goals and values (7).

NS reflects a heritable bias in the initiation or activation of appetitive approach in response to novelty, approach to signals of reward, active avoidance of conditioned signals of punishment, and escape from unconditioned punishment. HA involves a heritable bias in the inhibition of behavior in response to signals of punishment and frustrates no reward. RD is characterized by sentimentality, social sensitivity, attachment, and dependence on approval by others. P reflects a heritable bias in the maintenance of behavior despite frustration, fatigue, and intermittent reinforcement (8, 9).

In theoretical perspective, character of personality is related to self-concept schemas, aims, and values. Through character factors, SD is defined by self-comprehension as an independent person who has integrity, respect, effectiveness, leadership, and hope. CO is defined by self-apprehension as an integral part of human society that includes social feeling and compassionate. ST reflects the extent to which people conceptualize themselves as an integral part of the universe as a whole. ST individuals are described as judicious, insightful, spiritual, unpretentious, and humble (10).

Matsudaira and Kitamura presented that immaturity in any temperament can be a risk factor for depression. They performed TCI and criteria for hospital anxiety and depression (HAD) scale, on 541 Japanese scholars, and found that depression can be predicted through low scores in RD, P, SD, CO, and ST. Specific anxiety can be also predicted by higher score in NS, HA, P, ST, and lower scores in SD. Immaturity in SD can be a risk factor for negative emotion as well (11).

When compared with healthy individuals, bipolar patients achieve higher scores on NS (12-14), HA (12, 14-16) and RD (16), and lower scores on P (13, 15), and SD (14, 16). In bipolar disorder, higher scores on HA were also associated with early onset of disease and fewer suicide attempts (16, 17).

It is mentioned that HA and NS are high in BIID patients with mixed period (18).

Strakowski found that higher scores in NS predict weak prognosis in manic patients (18). In the study, which was carried out by Osher et al. it was shown that compared to the controls the score of persistence was lower in patients with BIID (15). Also, findings indicate that depression is associated with a decrease in NS and SD, and increase in HA and RD (19).

It has been reported that a decrease in CO in TCI is seen in Beck Depression Inventory patients that measure helpfulness and being passionate (14, 16, 20, 21). Young reported that in BIID patients and depression patients both before and after recovery episode, an increase in HA and NS without RD change was observed (12).

Based on previous studies, our main hypothesis was that bipolar patients would differ from MDD patients on some personality traits. If personality structure is different, then knowing the manner in which personality traits manifest themselves in BIID patients compared to MDD patients might help to disentangle the factors implicated in the etiology and clinical course of disorder and assist in optimizing research and treatment planning.

## Materials and Methods

This analysis included a sample of 45 BIID patients (30 men and 15 women), and 47 MDD patients (30 women and 17 men), aged 18-55 years who were diagnosed by psychiatric assessment based on axis I of Diagnostic and Statistical Manual of Mental Disorders, Fourth Edition (DSM-IV) diagnosis by Structured Clinical Interview for DSM-IV Axis I Disorders inventory. All subjects were recruited over 3 months by purposive sampling method. The two groups were matched regarding some demographic factors (gender, job type, marital status, and level of education). Exclusive criteria included psychotic disorders, mental retardation, and history of brain damage.

Personality traits were assessed using the TCI invented by Cloninger in 1994 (5). It has been extensively evaluated in Iranian subjects by Kaviyani and Pournaseh in Tehran and Shiraz (22). The TCI is a self-report questionnaire composed of 240 true/false items designed to measure individual difference on seven dimensions of temperament and character. Patients answer the questions of this inventory based on emotional reaction, feeling, favorites, attitudes, goals, and values. In other word, subjects describe themselves by their answers (5, 22).

***Data analysis***

Descriptive analysis of data was done using the statistical software SPSS for Windows (version 16.5; SPSS inc., Chicago, IL, USA). The results are reported as mean, maximum, and minimum for continuous variables. Student’s independent t-test was used to examine significant difference between the TCI personality traits of the subjects. The dependent variables of research were temperament and character scores. The independent variables were depression and BIID. The level of significant was set at 5% (p < 0.05 was considered as statistically significant).

## Results

The average age of participants was 33 (±10) years, education levels of majority of them had diploma and the job of most was a government employee for men and home maker for women.

According to table 1, by comparing the personality factors, MDD group had higher score in RD, P, and HA. But, only difference in RD score was statistically significant. On the other hand, mean CO, ST, SD, and NS scores were higher in BIID group and differences in NS, ST, and SD were statistically significant.

## Discussion

Regarding the temperament dimensions, there was a significant difference in terms of NS between MDD and BIID. This has been confirmed by some previous authors (18, 21). According to the role of dopaminergic system in NS behaviors, and according to the fact that increased level of dopamine in cerebrospinal fluid is seen in BIID patients with hypomanic episodes, some authors concluded that increased levels of dopamine is the reason of high scores of NS in patients with BIID. It could be hypothesized that in contrast to BIID patients, MDD patients are not sensation seekers because of their hopelessness (12, 18, 21, 23).

RD of BIID group showed a significant difference compared to MDD group. Bipolar patients are sensitive to social rewards when they experience hypomania and also as one can see they just fall into depression episode for a small annoying stimulus. These findings are in contrast to Engestrom’s results, but are in line with Osher’s results (15, 16).

Turning attention to the character traits, SD and ST had significant difference between MDD and bipolar patients. Low score in SD of MDD group can be a risk factor for committing suicide. These people do not feel prepared to cope with problems and feel frustrated. With this assumption, difference in the low score in SD can show a much important role of environmental factors and maybe traumatic experiences during childhood of MDD patients.

**Table 1:** Comparison of personality factors between major depressive disorder patients and those with bipolar II disorder

**Factors**	**Groups**		**P-values**
	**MDD** † ** group**	**Bipolar II disorder group**	
**Novelty seeking**	07.87 ± 3.20	10.29 ± 2.20	0.001
**Harm avoidance**	10.13 ± 2.90	09.73 ± 2.20	0.470
**Reward dependence**	08.82 ± 2.00	06.93 ± 1.90	0.001
**Persistence **	01.89 ± 1.00	01.56 ± 0.80	0.075
**Self-direction**	11.47 ± 4.10	13.42 ± 3.38	0.016
**Self-transcendence**	09.74 ± 2.11	11.64 ± 1.90	0.001
**Cooperativeness**	15.34 ± 2.30	16.09 ± 2.15	0.118

Data are presented as mean ± SD;

† Major depressive disorder

High score in ST in bipolar patients can show adaptive relationship with situations. They have more compliance in any way and search novelty in everything when compared with MDD patients. This is in complete opposition to MDD group where patients are serious critics of the world and its situation overlooking every new thing make them to be bored, numbing, tired and flat. Evans reported some of the findings in bipolar group as well (14).

In spite of the cross-sectional design of our study, this research faced some limitations such as small sample size, which can decrease statistical power of the study and inhibit findings for generalization. According to Cloninger’s theory, NS decreases with aging, so the results could not generate be generalized to other age groups. We used a self-report inventory only and maybe there is uncontrolled bias in these instruments.

We suggest a treatment plan design for disorders upon personality traits and using TCI tool for screening people who are susceptible to psychiatric disorders as a secondary prevention goal. 

## Notes

**Declaration of interest:** None.
